# The Culture of Bottle-feeding

**DOI:** 10.4103/0970-0218.55293

**Published:** 2009-07

**Authors:** Vivek Lal, Sanjay K Rai

**Affiliations:** Centre for Community Medicine, All India Institute of Medical Sciences, New Delhi – 110 029, India

## Introduction

The art of infant feeding is a blend of biology and culture and has been shown by several studies to be under strong influence of the socio-cultural milieu, which could be detrimental to the health of both mother and child. Blanket health messages to the community on infant feeding may fail to account for this cultural determinant. The following report is a case in point.

## Case Report

**Figure 1:**
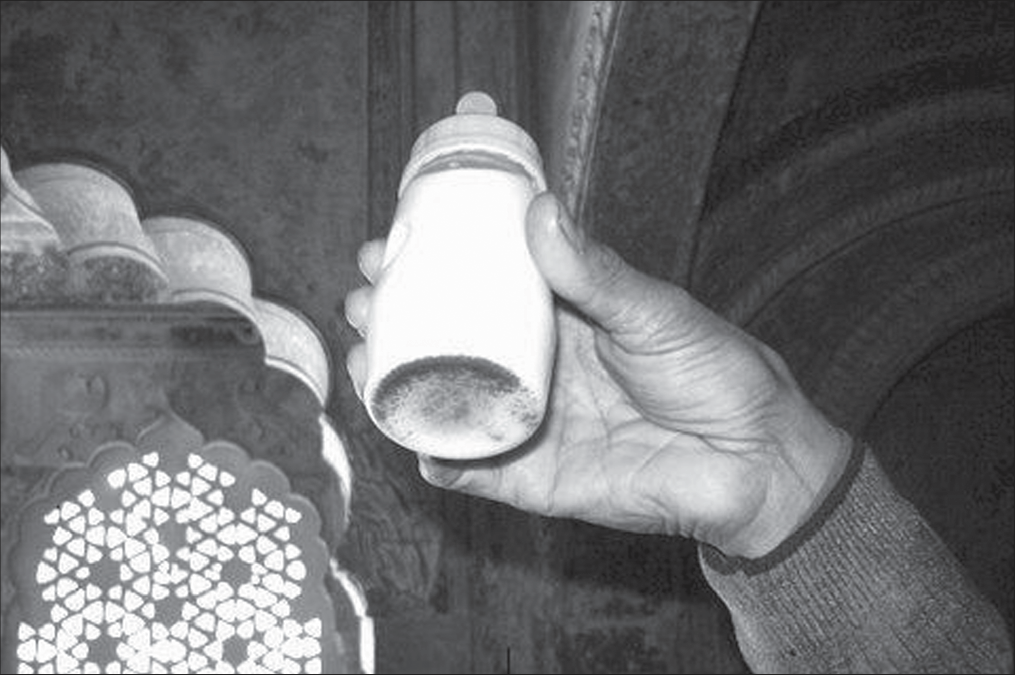
Feeding bottle with milk and charcoal

The case being reported is that of a one-month old child brought for a check-up at the Pediatrics OPD (*Chhatri*) at the Comprehensive Rural Health Services Project (CRHSP), All India Institute of Medical Sciences at Ballabgarh, Haryana. The child was suffering from diarrhea and vomiting without dehydration and was brought by his grandmother. It was learnt that the mother on observing that her milk secretion was inadequate, had begun to bottle feed the child in addition to breastfeeding, twice a day. On examining the bottle (which did not have the cap to cover the nipple and was instead wrapped in a cloth), it was found that it had black particles settled down at the base [Figure 1]. They were identified by the grandmother as *raakh* (charcoal) and reasoned that the milk was white in color: in order to avoid the *uppari hava* (evil eye), it needed to be given a black tinge.

## Discussion

To achieve optimal growth, development and health, the World Health Organization (WHO) recommends that infants should be exclusively breastfed for the first six months of life. Thereafter, to meet their nutritional requirements, infants should receive adequate and safe complementary foods while breastfeeding continues up to two years of age and beyond. It is further recommended that a feeding bottle with a nipple should not be used at any age.(1)

In addition to the risks posed by not having breast milk's protective qualities, breast milk substitutes and feeding bottles (where it is often difficult to sterilize the nipple properly), in particular, carry a high risk of contamination that can lead to life-threatening infections in young infants. The feeding bottle is an important factor in the infamous malnutrition- infection cycle, often reported to be a major cause of infant and child mortality.

Several studies provide interesting insights into the socio-cultural aspects of breast-feeding. It is evident that there is a strong tradition of breastfeeding in India, especially in the rural areas. But owing to the rural-urban migration of the poor, the increased interaction of this group with the concomitants of modernization including mass media communication networks, access to job opportunities, commercial products and their availability, has set into process a consumerist behavior based on imitation rather than need or rational decision-making.(2)

Universal exclusive breastfeeding (EBF) for the first six months could reduce infant mortality by 13%. Despite this knowledge, EBF rates remain low.

Lessons to learn: There is no reason for complacency on the part of health professionals. Each child and the family to which the child belongs need to be seen as an individual and unique case. Since a large number of women in our country are already traditionally inclined towards breastfeeding; merely pointing out the advantages of breastfeeding in general may be inadequate.(3) The need is to identify specific problems in the local community: this is essential for instituting health education. Detrimental practices should be identified and efforts should be concentrated on rectifying these. Child feeding problems are often rooted in cultural beliefs and practices that do not match the biologically based needs of mother and child. Leaders who understand the role of culture in feeding will be able to provide more culturally sensitive support and information to mothers.(4)

A study conducted in Uganda found that training of community- based peer counselors to support EBF was both feasible and accepted by communities.(5) Another study conducted in Antenatal clinics setting in South Africa demonstrated that a simple and inexpensive counseling intervention along with home support had a great effect on EBF rates.(6)

It is important that every opportunity of contact of health personnel with care-givers should be taken to counsel on infant feeding. All cadres of health personnel should be provided training in accordance to and suitable for implementation at local level. They must be followed up and supported in their work. Further, positive deviants must be identified in every community setting and others should be motivated to adopt the approach. The program can be evaluated through focus group discussions with health care providers and child care-givers.
